# Gene filtering strategies for machine learning guided biomarker discovery using neonatal sepsis RNA-seq data

**DOI:** 10.3389/fgene.2023.1158352

**Published:** 2023-04-11

**Authors:** Edward Parkinson, Federico Liberatore, W. John Watkins, Robert Andrews, Sarah Edkins, Julie Hibbert, Tobias Strunk, Andrew Currie, Peter Ghazal

**Affiliations:** ^1^ Department of Computer Science and Informatics, Cardiff University, Cardiff, United Kingdom; ^2^ Project Sepsis, Systems Immunity Research Institute, Cardiff University, Cardiff, United Kingdom; ^3^ Wesfarmers Centre of Vaccines and Infectious Diseases, Telethon Kids Institute, Perth, WA, Australia; ^4^ Medical School, University of Western Australia, Perth, WA, Australia; ^5^ Centre for Molecular Medicine and Innovative Therapeutics, Murdoch University, Perth, WA, Australia; ^6^ Neonatal Directorate, Child and Adolescent Health Service, Perth, WA, Australia

**Keywords:** neonatal sepsis, machine learning with RNA-seq, transcriptomic sepsis biomarkers, independent gene filtering, gene signature stability

## Abstract

Machine learning (ML) algorithms are powerful tools that are increasingly being used for sepsis biomarker discovery in RNA-Seq data. RNA-Seq datasets contain multiple sources and types of noise (operator, technical and non-systematic) that may bias ML classification. Normalisation and independent gene filtering approaches described in RNA-Seq workflows account for some of this variability and are typically only targeted at differential expression analysis rather than ML applications. Pre-processing normalisation steps significantly reduce the number of variables in the data and thereby increase the power of statistical testing, but can potentially discard valuable and insightful classification features. A systematic assessment of applying transcript level filtering on the robustness and stability of ML based RNA-seq classification remains to be fully explored. In this report we examine the impact of filtering out low count transcripts and those with influential outliers read counts on downstream ML analysis for sepsis biomarker discovery using elastic net regularised logistic regression, L1-reguarlised support vector machines and random forests. We demonstrate that applying a systematic objective strategy for removal of uninformative and potentially biasing biomarkers representing up to 60% of transcripts in different sample size datasets, including two illustrative neonatal sepsis cohorts, leads to substantial improvements in classification performance, higher stability of the resulting gene signatures, and better agreement with previously reported sepsis biomarkers. We also demonstrate that the performance uplift from gene filtering depends on the ML classifier chosen, with L1-regularlised support vector machines showing the greatest performance improvements with our experimental data.

## 1 Introduction

Supervised machine learning (ML) analysis of gene expression data is a widely used AI approach to derive informative gene subsets with potential utility as diagnostic and prognostic clinical biomarkers (Liu et al., 2014; Lin et al., 2017; Zhang et al., 2021). These ML based feature selection algorithms have the power to both eliminate redundant genes, and identify relevant genes that both discriminate between clinical conditions of interest, and that generalise over the relevant patient populations (Mahendran et al., 2020). These techniques are referred to as ML gene selection or simply gene selection throughout this work. To date, the majority of validated sepsis biomarkers have been derived from microarray gene expression data (Smith et al., 2014; McHugh et al., 2015; Sweeney et al., 2015). In recent years RNA-Seq has largely replaced microarray as the preferred technology for generating gene expression data, given the higher resolution and decreasing cost of sequencing (Wang et al., 2009; Stark et al., 2019). The combination of ML gene selection with richer RNA-Seq data presents the opportunity to discover previously unidentified sepsis biomarkers. ML classification algorithms are however highly sensitive to any feature data characteristic, regardless of scale, that may differ between experimental groups, and will exploit these data characteristic differences in gene selection. Systematically adding noise to feature data has been shown to significantly degrade classification performance across a range of ML classification algorithms (Zhu and Wu, 2004; Hasan and Chu, 2022). It is therefore desirable to minimise this interference to ensure patterns detected have biological relevancy. To date the impact of applying independent filtering and pre-processing strategies to eliminate noise on the performance of ML approaches to biomarker discovery with RNA-Seq data has not been fully investigated.

Here we consider two sources of noise inherent in RNA-Seq data that may negatively impact ML gene selection. Firstly low read counts. Genes with consistently low read count values across all replicates may be technical or biological stochastic artefacts such as the detection of a transcript from a gene that is not uniformly active in a heterogeneous cell population or as the result of a transcriptional error (Wagner et al., 2013). Below some count threshold, genes with low read counts are subject to greater dispersion (variability) with greater false negatives (zero inflation) and false positives (outliers) that are not representative of true biological differences related to the condition of interest. The filtering out of low read count genes from RNA-Seq data in differential expression analyses is reported to improve detection of differentially expressed genes by reducing the impact of multiple testing corrections (Bourgon et al., 2010). A wide variety of approaches to filtering low count genes have been proposed, the most common being maximum-based filters, where genes with a maximum normalised count over all samples below a threshold *t* are filtered out (Rau et al., 2013). At a lower threshold *t*, the number of genes retained after filtering is expected to be more variable between samples, given differences in low count noise between samples. As *t* increases, the number of genes retained is expected to converge as the read counts increasingly represent the true biological signal, which is more consistent across samples (Koch et al., 2018). To avoid setting arbitrary thresholds, multiple authors have proposed approaches to setting the filter threshold based on the data. Rau et al. (2013) determine the optimal threshold based on maximising the similarity of expression between samples. Love et al. (2014) implement gene filtering in the R package DESeq2 to maximise the number of genes found to be significantly differentially expressed based on a user-specified target false discovery rate (FDR). Deyneko et al. (2022) derive a sample specific threshold by separately modelling the random (low count noise) and true biological signal, and subtracting the low count noise component from the reads of each sample. Zehetmayer et al. (2022) propose an adaptive approach, testing the impact of multiple filters and selecting the filter that maximises the power of a hypothesis test in the given data set. In all these approaches, the goal of independent gene filtering is to reduce the impact of multiple testing correction in gene by gene hypothesis testing. In a multivariate supervised learning setting, it is well known that features may have significant effects in combination despite having weak effects individually (Guyon and Elisseeff, 2003). This raises the question of whether and how low count filtering should be performed. Filtering out genes based on arbitrary thresholds risks discarding valuable biomarkers that may have discriminate power in combination with other genes and provide useful insights into underlying biology.

A second source of noise are influential outlier read counts. Relatively high read counts occurring in only a very small number of samples relative to the size of each patient group are unlikely to be representative of the general population and a result of biological heterogeneity and technical effects. These gene values may bias feature selection algorithms as they discriminate between examples in a training set, leading to model overfitting. Such influential outliers may be the result of natural variation between individuals or they may have been introduced during sample preparation. cDNA libraries require PCR amplification prior to sequencing to achieve sufficient sequence depth. This clonal amplification by PCR is stochastic in nature; different fragments may be amplified with different probabilities. This leaves the possibility of outlier read counts having resulted from bias introduced by amplification, rather than biological differences between samples (Fu et al., 2018; Stark et al., 2019). As with low count genes, in the context of differential expression analyses, hypothesis testing based on the negative binomial distribution, extreme outlier read counts can have a disproportionate effect on the results, increasing false positives and false negatives, and inflating the observed association between particular genes and the condition of interest (Mangiola et al., 2021). Multiple approaches to identify and remove outliers have been proposed in this context (Love et al., 2014; Brechtmann et al., 2018; Mangiola et al., 2021).

Despite significant work in the area of differential expression analysis, to our knowledge, there is a lack of analysis of the impact of gene filtering on downstream ML analysis, specifically in gene selection for biomarker discovery. Although multiple software pipelines have been developed to aid researchers in conducting this type of analysis using high-throughput sequence data (Chiesa et al., 2018; Goksuluk et al., 2019; Dag et al., 2022), gene filtering features are limited to removing low count and low variance genes and typically require users to provide their own arbitrary thresholds. Previous authors provide little justification for the use of these filters, nor do they provide recommended thresholds or guidance on how these might be adjusted to the ML algorithm being employed.

Evaluation of the impact of gene filtering in ML gene selection requires a success measure equivalent to the number of significant genes identified by a series of hypothesis tests in the differential expression analysis setting. In the task of biomarker discovery, the chosen set of genes effectively form an hypothesis on the underlying biological mechanisms of disease. The ability of the selected genes to discriminate between disease states is of primary importance if the biomarkers are to be translated into a clinical setting. Perhaps equally important to their predictive power is the stability of the chosen gene set in the event of changes to the training dataset (Jurman et al., 2008; Sechidis et al., 2019). An unstable gene selection procedure can be thought of as one where small changes to the training data result in large changes to the chosen gene set (Nogueira et al., 2018). We propose therefore that the appropriate way to evaluate the impact of gene filtering is to measure the relative classification performance of a derived gene set *and* the stability of the gene set to perturbation of the training data with and without gene filtering employed. A stability measure quantifies the similarity of a group of gene sets, often giving a value between zero and one, where zero implies a random choice of genes in each set, and one implies identical sets. A wide variety of stability measures have been proposed, and a comprehensive review is provided by Bommert et al. (2017) and Nogueira et al. (2018). Where existing biomarkers have been validated, as in the case of sepsis, an additional measure is the level of agreement of a new gene set with existing validated biomarkers.

In this article, we examine the impact of gene filtering on the performance and stability of three common ML algorithms for sepsis biomarker discovery using bulk RNA-Seq data. The motivation of this article is to assess the need to perform independent gene filtering in preparation for a ML analysis, the implications of not doing so, and the impact of gene filtering on the downstream classification performance and stability to changes in experimental data. We provide practical guidelines on the gene filtering steps needed in ML analysis of RNA-Seq data absent in the existing literature. The rest of this article is organised as follows: In the Materials and Methods section we summarise the data processing pipeline, the datasets used and the nature of the gene filters and ML classifiers applied. The Results and Discussion section demonstrates the impact of gene filtering on feature selection and the classification performance of the resulting gene sets, as well as their stability, and agreement with previously validated sepsis biomarkers. Finally, the Conclusion summarises the rationale for gene filtering prior to ML gene selection and outlines areas for future investigation.

## 2 Materials and methods

This section introduces the datasets used in this work and provides a brief theoretical background to the gene filter methods and supervised ML approaches employed.

### 2.1 Data processing pipeline overview

**FIGURE 1:**
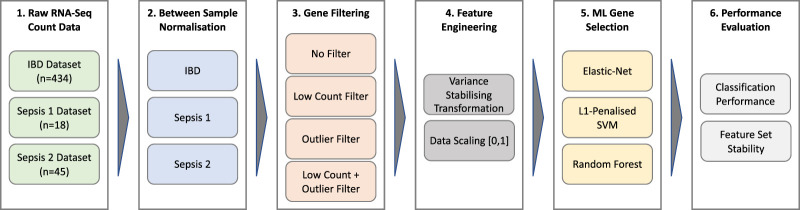
Data processing and analysis pipeline.

A schematic overview of the data processing and analysis pipeline used in this work is depicted in Figure 1. A detailed description of each stage of the pipeline is provided in the following sections.

### 2.2 Test datasets

The impact of gene filtering on ML performance is demonstrated using three experimental bulk RNA-Seq datasets. To the best of our knowledge, there are a limited number of RNA-Seq datasets profiling sepsis patients, and those that exist contain a small number of samples. We therefore conduct our analysis using two relatively small cohorts of neonatal infants containing sepsis cases, supplemented by a publicly available dataset of inflammatory bowel disease patients containing a far larger number of samples, to assess generality as well as increase confidence in the results.

#### 2.2.1 Inflammatory bowel disease (“IBD”) dataset

The IBD data is taken from the recent study by Nowak et al. (2022) who deposited 590 pediatric and adult patient samples in the EMBL-EBI ArrayExpress database (E-MTAB-11349). The data used in this work include the raw count data from 434 patients recruited at six gastrointestinal clinics across Europe between 2012 and 2016, with 167 patients diagnosed with ulcerative colitis and 267 controls. 49% of the patients are female and they range in age from 3 to 79. Details of the sequencing whole-blood RNA for this cohort using the Ion Torrent sequencing platform are given by Nowak et al. (2022).

#### 2.2.2 Neonatal sepsis datasets

The first neonatal sepsis dataset (referred to as Sepsis 1 below) derives from a 2020 study by Ng et al. (2020) of 18 very preterm neonatal infants, deposited in the NCBI GEO omnibus gene expression database (GSE138712). Infants are classified based on a positive blood culture and elevated C-reactive protein concentrations as having confirmed late onset sepsis (*n* = 5), clinical late onset sepsis (*n* = 4) or no late onset sepsis (*n* = 9). Suspicion of sepsis in premature infants has a low threshold and there is a good possibility that they will not have sepsis. For the purposes of this work we have tested this assumption using neonatal sepsis biomarkers described by Smith et al. (2014), hereafter referred to as the “Sep3” gene signature. Accordingly, all clinical late onset sepsis cases are considered to have no late onset sepsis, with the exception of a single sample, classified as confirmed sepsis based on clear discrimination from PCA analysis using neonatal sepsis biomarkers and illustrated in Supplementary Figure S1. The retention of the clinical late onset sepsis group and allocation between confirmed and no sepsis is further justified by the need maintain the sample size and class balance to aid the performance of ML classification algorithms in what is already a very small dataset. Details of the sequencing whole-blood RNA procedure are provided by Ng et al. (2020).

The second sepsis dataset (referred to as Sepsis 2 below) derives from a cohort of 45 very preterm infants recruited from the neonatal intensive care unit of King Edward Memorial Hospital, Perth, Australia, a sub-study of The PROTECT Trial (Simmer et al., 2016), a pragmatic randomised placebo-controlled clinical trial evaluating the effectiveness of intravenous pentoxifylline for improving long-term outcomes in preterm infants with late onset sepsis or necrotising enterocolitis. The PROTECT study was approved by the institutional Human Research Ethics Committee at King Edward Memorial Hospital, Perth, Australia (RGS0000002684). Written, informed consent was obtained by the Principal Investigator or delegate from the parent(s) prior to study participation. Infants with suspected late onset sepsis ( >72 hours after birth) were enrolled in the sub-study between 2016 and 2019.12 of the 45 infants were classified as having confirmed late onset sepsis in the event of a positive blood culture and the causative pathogen identified, two or more serial CRP measurements of > 20 mg/L within 72 h of blood culture and having been treated with antibiotics for five or more consecutive days. The remaining 33 infants are classified as no late onset sepsis, with negative blood culture, all CRP measurements below 20 mg/L and not more than 3 days of treatment with antibiotics. A peripheral whole blood sample was taken near the time of blood culture sampling for suspected late onset sepsis and prior to administration of study treatment. RNA was stabilised in PAX and stored at −80C until extraction. Total RNA quality and quantity was assessed using Agilent 4,200 TapeStation and a High Sensitivity RNA kit (Agilent Technologies). 75–100 ng of Total RNA with a RIN value >7 was depleted of ribosomal RNA using the NEBNext^®^ Globin and rRNA Depletion Kit (Human/Mouse/Rat) (New England BioLabs, NEB) and the sequencing libraries were prepared using the NEB^®^ Ultra™ II Directional RNA Library Prep Kit for Illumina^®^ (NEB). The steps included RNA fragmentation and priming, first strand cDNA synthesis, 2nd strand cDNA synthesis, adenylation of 3′ ends, adapter ligation, PCR amplification (16-cycles) and validation. The manufacturer’s instructions were followed. The libraries were validated using the Agilent 4,200 TapeStation and a DNA1000 tape (Agilent Technologies) to ascertain the insert size, and the CLARIOstar^®^ (BMG Labtech) was used to perform the fluorometric quantitation. Following validation, the libraries were normalized to 3nM, pooled together and sequenced using a 100-base paired-end (2 × 100bp PE) dual index read format on the Illumina^®^ NovaSeq™6000 according to the manufacturer’s instructions. Paired-end reads from Illumina sequencing were trimmed with Trim Galore (Babraham Bioinformatics Group, 2019a) and assessed for quality using FastQC (Babraham Bioinformatics Group, 2019b), using default parameters. Reads were mapped to the human GRCh38 reference genome using STAR (Dobin et al., 2013) and counts were assigned to transcripts using featureCounts (Liao et al., 2014) with the GRCh38.96 Ensembl gene build GTF. Both the reference genome and GTF were downloaded from the Ensembl FTP site (Ensembl, 2021).

### 2.3 Between sample normalisation

Raw read counts for each dataset are scaled using the median-of-ratios method described by Anders and Huber (2010) (often referred to as “normalisation” in bioinformatics literature) to account for systematic differences between samples resulting from technical factors, namely sequence depth (total number of aligned reads) and sample composition (relative proportion of transcripts for a given number of reads). These normalised read counts allow comparison of the relative expression level for each gene between samples. Filtering to identify genes with relatively low and extreme outlier read count genes is conducted using these normalised counts as described below.

### 2.4 Gene filtering

To demonstrate the impact of filtering low count and influential outlier genes on feature selection in a supervised ML setting, we apply two widely used filtering methods to each datasets. For the remainder of this work, the term gene is used as a shorthand to refer to an RNA transcript with a unique EBI Ensembl or NCBI RefSeq accession number; the level at which filtering is implemented.

#### 2.4.1 Low count genes

To filter out low expression genes, we apply the data-based maximum filter approach proposed by Rau et al. (2013) and demonstrated to be superior to other maximum based filters for filtering out low count noise in a differential expression analysis setting. This approach derives a filter threshold *t* that maximises the similarity between samples using the Jaccard index (Jaccard, 1901). The vector of read counts for all genes in a given sample *j* with experimental condition *c*(*j*) is given by **s**_*j*_. **s**_*j*_ is binarised for a given threshold *t* (**s**_*j*_ > *t* is 1, otherwise 0). The Jaccard similarity index between the binary vectors for each pair of samples *j* and *j*′ from the same experimental condition (*c*(*j*) = *c* (*j*′)) is given by Eq. 1.

$$\begin{array}{c} J_{S}\left(s_{j},s_{j^{\prime }}\right)=\frac{s_{j}⋂s_{j^{\prime }}}{s_{j}⋃s_{j^{\prime }}} \end{array}$$ (1)

The optimum threshold t* is defined as the threshold that maximises the similarity between samples, corresponding to the maximum average Jaccard index over all pairs in each experimental condition, as in Eq. 2.

$$\begin{array}{c} t^{*}=\underset{t}{\arg\max}\text{ mean }\left\{J_{S}\left(s_{j},s_{j^{\prime }}\right):j<j^{\prime }\text{ and }c\left(j\right)=c\left(j^{\prime }\right)\right\} \end{array}$$ (2)

#### 2.4.2 Influential outlier genes

To identify sample gene pairs with influential outlier read counts, we implement the approach used in DESeq2 (Love et al., 2014) that uses Cook’s distance (Cook, 1977a). Briefly, Cook’s distance is a measure of influential points in a generalised linear regression model (GLM). In the context of the negative binomial distribution most frequently used to model RNA-Seq count data, Cook’s distance is given by Eq. 3, where *R*_*ij*_ is the Pearson residual of sample *j*, a measure of the extent to which an observed value differs from the predicted value in the model; *h*_*ii*_ is the leverage of each count, which can be thought of as the weighted distance the values of the independent variables vary from the mean, *τ* is an over dispersion parameter that is set to 1 in the GLM, and *p* is the number of parameters. The Cook’s distance *D*_*ij*_ is the scaled distance that the coefficient vector *β*_*i*_ of the GLM for gene *i* would change if the sample *j* were removed and the model refit. Counts with larger values of *D*_*ij*_ therefore have a greater influence on the model parameters.

$$D_{ij}=\frac{R_{ij}^{2}}{\tau p}\frac{h_{jj}}{{\left(1-h_{jj}\right)}^{2}}$$ (3)

Influential outliers are defined by transforming the values of *D*_*ij*_ to points on the *F* (*p*, *m* − *p*) distribution where the *p* is the number of model parameters and *m* is the number of samples, and defining a threshold by an arbitrary quantile *q* (Cook, 1977b). In this work *q* is set to 0.95, and a gene is filtered out if an influential outlier read count is present in one or more samples.

Following filtering, the filtered genes are removed from the raw count data and the raw counts are re-normalised by the median-of-ratios method.

### 2.5 Feature engineering

The skewed and heteroskedastic nature of RNA-Seq count data means that certain machine learning algorithms, in particular those such as support vector machines employing distance based measures, may be disproportionately influenced by the genes with the highest mean counts, due to the larger variance (absolute distances) between samples. To overcome this potential issue, the median-ratio scaled data is further pre-processed with the variance stabilising transformation described by Love et al. (2014) to reduce both the skewness and the mean-variance relationship and scaled to values between 0 and 1 to ensure all genes are on a comparable scale.

### 2.6 Gene selection with supervised machine learning

**FIGURE 2:**
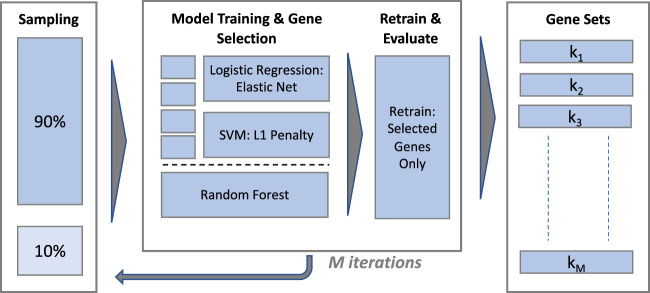
Gene selection performance and stability evaluation.

Gene selection is performed using supervised ML classification algorithms with embedded feature selection and computationally efficient implementations in R, henceforth referred to as classifiers or models interchangeably. The overall scheme for model training is illustrated in Figure 2.

#### 2.6.1 Choice of machine learning classifiers

The classifiers selected are elastic net regularised logistic regression (eNet) (Zou and Hastie, 2005; Friedman et al., 2010), L1 regularised Support Vector Machines using the LIBLINEAR library (L1-SVM) (Fan et al., 2008) and Random Forest (RF) (Breiman, 2001). These methods are chosen for several reasons. Firstly because they are widely known and frequently used in gene selection applications (Mahendran et al., 2020). Secondly because each of these ML methods applies fundamentally different principles to fit the data, including linear (eNet, L1-SVM) and non-linear (RF) models, allowing us to investigate whether gene filtering approaches are applicable over a diverse set of ML approaches. Thirdly and perhaps most importantly because feature selection is embedded within these three methods. eNet and L1-SVM implicitly perform feature selection as a result of the L1 penalty shrinking the coefficients of the least important features to zero during the optimisation. In the case of Random Forest, the relative importance of features can be calculated following model training, and features ranked by importance. Other machine learning approaches without this property of embedded feature selection would require either a gene selection filter method to be applied prior to training the classifier, or a wrapper method such as recursive feature elimination (Guyon et al., 2002) to be applied during model training. Both these alternatives are deemed to complicate the analysis unnecessarily, and in the case of recursively searching for an optimum feature set, to increase the computational complexity so as to make the analysis impractical given the high dimensionality of the datasets.

#### 2.6.2 Model training and gene selection

The procedure used to train each classifier and extract a gene set is illustrated in the central panel of Figure 2. The eNet and L1-SVM models are trained with a range of values of their respective regularisation hyperparameters in a k-fold cross validation scheme (k = 10 for the IBD dataset and ‘leave-one-out’ for the sepsis datasets) to provide a validation set for classification performance evaluation. Hyperparameter values are selected to yield a gene set of similar size for each model, targeting 30 genes for the IBD and Sepsis 2 datasets and 10 genes for the Sepsis 1 dataset, given the smaller number of samples in this case. Fixing the model hyperparameters to yield gene sets of a similar size is important to ensure gene set stability measures are broadly comparable between the models. A final model is trained on the full dataset, without cross validation, using the selected hyperparameter values, and the set of selected genes identified. The model predictions on the cross validation set at the chosen hyperparameter values are used to evaluate the classification performance^1^.

The RF classifier is implemented with fixed hyperparameters (1,000 trees and $\sqrt{p}$ randomly selected genes per tree, where *p* is the total number of genes in the training set). Feature importance based on maximum decrease in Gini coefficient is used to manually select the most discriminative genes with respect to the target condition (Bommert et al., 2020). The top 30 genes by feature importance are selected for the IBD and Sepsis 2 datasets, and the top 10 are selected for the Sepsis 1 dataset. A final model is retrained on the full dataset using only the selected features, and the predictions on the out-of-bag samples used to evaluate classification performance.

### 2.7 Performance evaluation

#### 2.7.1 Classification performance measurement

Classification performance is evaluated using the *F*_1_ score (Chinchor, 1992), a widely used metric for evaluating ML classification performance, in particular where the two experimental groups under consideration are of different sizes, as is the case here. *F*_1_ score is the harmonic mean of the Sensitivity (also known as Recall) and Positive Predictive Value (PPV, also known as Precision), as defined in Eq. 4, and gives a value in the range 0–1.

$$F_{1}=2\cdot \frac{Sensitivity\cdot PPV}{Sensitivity+PPV}$$ (4)

A baseline *F*_1_ score can be derived by assuming that a ‘dumb’ classifier predicts all patients to be the positive class (e.g., sepsis), in which case the *F*_1_ score simplifies to 2*p*/(*p* + 1), where *p* is the probability of a positive example in a given dataset.

#### 2.7.2 Feature stability measurement

To estimate gene set stability with and without gene filtering, training and gene selection was repeated on *M* 90% random stratified sub-samples of the unfiltered and filtered data (with *M* = 100), producing gene sets *k*_1_, *k*_1_, … ,*k*_*M*_ in each case.

In the context of transcriptomic biomarker discovery, given the high level of correlation between features, two feature sets may contain different genes that are however correlated as a result of related biological function. Feature selection algorithms may only select one of a number of correlated features, and therefore if these correlations are not taken into account, stability may be underestimated (Sechidis et al., 2019). The recently reported stability measure by Sechidis et al. (2019) was considered for this work, however was found to be computationally expensive to compute using our very high dimensional datasets. Thus, gene set stability over the *M* gene sets is calculated using the measure proposed by Zucknick et al. (2008) that meets the majority of desirable properties of a stability measure (Nogueira et al., 2018; Bommert, 2020), takes into account the correlation between genes in the dataset and is bounded between 0 and 1, aiding comparisons between datasets. The Zucknick stability over *M* gene sets is the mean pairwise correlation-extended Jaccard index between set *V*_*i*_ and *V*_*j*_, defined in Equation 5. The intersection term is supplemented by a correlation factor *C*(*V*_*i*_, *V*_*j*_) defined as the mean absolute correlation *R*_(*x*,*y*)_ of all genes selected in *V*_*i*_ with all genes selected in *V*_*j*_ but not in *V*_*i*_. *R*_(*x*,*y*)_ is thresholded on parameter *τ* = 0.5, to avoid computing the effect of a large number of weak correlations.

$$\begin{array}{c} \hat{\Phi }\left(Z\right)=\frac{2}{M\left(M-1\right)}\overset{M-1}{\underset{i=1}{\sum }}\overset{M}{\underset{j=i+1}{\sum }}\frac{|V_{i}\cap V_{j}|+C\left(V_{i},V_{j}\right)+C\left(V_{j},V_{i}\right)}{\left|V_{i}\cup V_{j}\right|}\\ \;\\ C\left(V_{k},V_{l}\right)=\frac{1}{\left|V_{l}\right|}\underset{\left(x,y\right)\in V_{k}\text{x}\left(V_{l}\backslash V_{k}\right)}{\sum }1\left(R_{\left(x,y\right)}>\tau \right)|R_{\left(x,y\right)}|\\ \;\\ 1\left(R_{\left(x,y\right)}>\tau \right)=\left\{\begin{array}{c} 1,\;\text{ if }R_{\left(x,y\right)}>\tau \;\\ 0,\;\text{ otherwise}\; \end{array}\right. \end{array}$$ (5)

## 3 Results and discussion

The experimental results describe how gene filtering impacts the classification performance and stability of the gene sets selected by the three supervised ML approaches (eNet, L1-SVM and RF), and evaluates the biological relevance of the resulting sepsis biomarkers in the context of previously validated gene signatures.

### 3.1 Filtered genes

The derived low counts maximum filter thresholds for the IBD, Sepsis 1 and Sepsis 2 datasets were calculated as t* = 13, t* = 11, and t* = 60 respectively. The corresponding number of genes filtered out by the low counts, influential outlier and combined low counts and influential outlier filters are given in Table 1. The notable differences in the original number of genes reflects the different sequencing depths and technologies used for these cohorts.

**TABLE 1:** Filtered genes.

	Original	Retained	Filtered	% filtered
***IBD***				
Low Count	22,656	18,278	4,378	19
Outlier	22,656	21,052	1,604	7
Low Count + Outlier	22,656	16,674	5,982	26
***Sepsis 1***				
Low Count	28,325	13,361	14,964	53
Outlier	28,325	28,111	214	1
Low Count + Outlier	28,325	13,173	15,152	54
***Sepsis 2***				
Low Count	55,574	20,606	34,968	63
Outlier	55,574	54,942	632	1
Low Count + Outlier	55,574	20,176	35,398	64

The three datasets illustrate the potential variability in the proportion of genes that may be removed by filtering. The Sepsis 1 and Sepsis 2 datasets have a higher proportion of genes with low counts with 53% and 63% of the genes removed by the low counts filter respectively, compared with only 19% for the IBD dataset. The IBD dataset has a proportionally higher number of genes with influential outlier values, with 7% of genes removed by the outlier filter.

### 3.2 Gene Selection Frequency of low count and outlier genes

**FIGURE 3:**
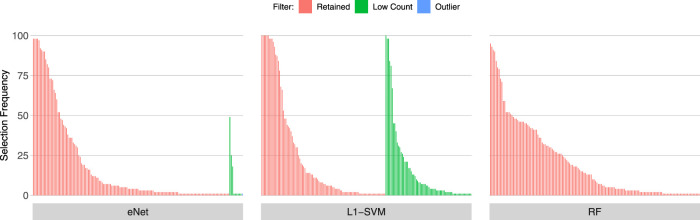
Gene selection frequency: IBD

**FIGURE 4:**
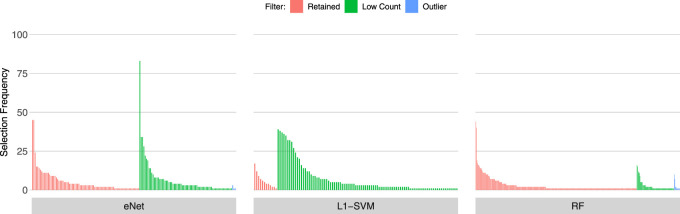
Gene selection frequency: Sepsis 1.

**FIGURE 5:**
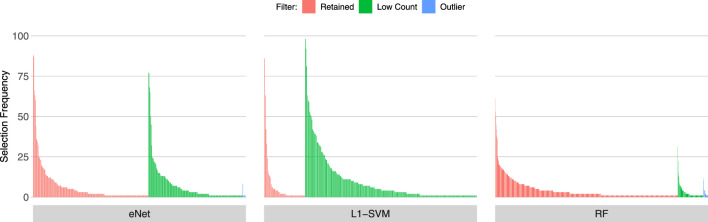
Gene selection frequency: Sepsis 2.

Figures 3, 4, 5 show the frequency of selection of all genes over *M* = 100 sub-samples of the three unfiltered datasets for each of the three ML gene selection approaches. In each plot, a bar on the horizontal axis represents a gene ID that is selected at least once over all sub-samples. The vertical axis gives the frequency that each gene is selected over 100 sub-samples. Genes selected by classifiers in the unfiltered dataset that would have passed both filters are labelled in red (Retained), selected genes that would have been filtered out by applying a low counts filter are labelled in green (Low Count), while those that would have been filtered out by the outlier filter are labelled in blue (Outlier).

It is striking that low count genes are selected in all datasets and by all classifiers, with the exception of RF with the IBD dataset. In both of the sepsis datasets, the majority of L1-SVM gene selections are low count genes, and low count genes represent the most frequently selected gene in the by eNet in the Sepsis 1 dataset and by L1-SVM in the Sepsis 2 dataset. RF appears to be the most robust to low count genes across all datasets. Influential outlier genes are also selected by the eNet and RF classifiers in both of the sepsis datasets, albeit at a lower frequency compared with low count genes likely due to the small number of genes identified as containing outlier read counts. These results clearly illustrate how, without prior filtering out of low count and influential outlier genes, ML gene selection is at risk of actively selecting non-informative and biasing genes that happen to be statistically useful in classifying the training examples, and as illustrated here, these non-informative genes can dominate the resultant patterns. This highlights the need for a considered and objective approach to filtering prior to conducting ML analysis.

### 3.3 Performance impact of gene filters

Table 2 outlines the *F*_1_ scores achieved on classifying the validation set (eNet and L1-SVM) or out-of-bag samples (RF) without filtering, with each filter applied independently and with the combined filters. The *F*_1_ scores for the L1-SVM and RF models are a mean value over ten repeated measures, and the mean and standard deviation are given, along with *p*-values of a *t*-test of mean *F*_1_ score for filtered vs. unfiltered cases, with the null hypothesis of equal means. The baseline *F*_1_ scores achieved by a dumb model predicting all examples as positive are 0.56 for IBD, 0.50 for Sepsis 1, 0.42 for Sepsis 2.

**TABLE 2:** Gene filter impact on classification performance.

	eNET	L1-SVM			RF		
Filter	*F*_1_	Mean *F*_1_	Sd	*p*-value	Mean *F*_1_	Sd	*p*-value
***IBD***							
None	0.885	0.703	0.005	—	0.703	0.007	—
Low Count	0.891	0.728	0.005	<0.001	**0.724**	0.011	<0.001
Outlier	0.895	0.725	0.002	<0.001	0.718	0.008	<0.001
Low Count + Outlier	**0.898**	**0.735**	0.002	<0.001	0.718	0.010	0.0014
***Sepsis 1***							
None	0.500	0.286	0.000	—	0.982	0.038	—
Low Count	**0.667**	**0.800**	0.000	—	**1.000**	0.000	0.168
Outlier	0.500	0.286	0.000	—	0.992	0.029	0.556
Low Count + Outlier	**0.667**	**0.800**	0.000	—	0.973	0.044	0.628
***Sepsis 2***							
None	0.762	0.671	0.071	—	0.944	0.021	—
Low Count	0.818	0.731	0.011	0.026	0.949	0.017	0.580
Outlier	0.762	0.709	0.036	0.154	0.949	0.017	0.580
Low Count + Outlier	**0.870**	**0.734**	0.015	0.021	**0.957**	0.000	0.082

The bold values are the highest scoring filter for each dataset.

All three classifiers achieve significantly higher than baseline performance on the IBD dataset, and classification performance is significantly improved by both the low counts and outlier filters. The performance scores for the Sepsis 1 dataset should be treated with some caution, given the small number of examples, with only 6 infants in the sepsis group. However, despite the eNet and L1-SVM models failing to perform better than the baseline in the unfiltered data, filtering again appears to improve performance. RF performance appears unchanged by gene filtering. Nevertheless, it can be said that removing over 50% of the genes in the Sepsis 1 dataset has not detrimentally impacted RF performance (all *p*-values are much greater than 0.05). Similarly, in the Sepsis 2 dataset, all models achieve above baseline performance, and the performance of eNet and L1-SVM is significantly improved by low count filtering and not negatively impacted by outlier filtering. Again RF performance is not significantly impacted by filtering. These results are consistent with previous studies on the impact of noise in feature data on machine learning classification performance. Zhu and Wu (2004) and Hasan and Chu (2022) both demonstrate that ML classification performance is materially improved by systematic removal of noise in feature data. By removing non-informative and biasing genes, the risk that such genes are used to construct a model is reduced, resulting in models that are better able to generalise to unseen patient data. In short, gene filtering reduces the risk of overfitting.

The consistent performance improvements across all three datasets using the eNet and L1-SVM models also indicate that gene filtering strategies are relevant to datasets with very different sample sizes (*n* = 434 for IBD compared with *n* = 18 for Sepsis 1), including datasets with very small sample sizes.

Comparing the performance of the three classifiers, in the Sepsis 1 and Sepsis 2 datasets, where 50%–60% of genes are removed by the low counts filter, RF achieves significantly better classification performance than eNet and L1-SVM, in particular where no filtering is applied. This result is consistent with the findings of (Hasan and Chu, 2022) that RF achieves relatively higher classification performance than a range of other classifiers in the presence of high levels of feature noise over a range of datasets.

One point of caution in interpreting these results: although the dimensionality of the datasets have been significantly reduced by gene filtering, and classification performance is improved on the validation set in the majority of cases, the *F*_1_ scores should be interpreted in relative terms only. The risk remains that these classifiers are somewhat over fit to these relatively small datasets, and further work is required using a larger number of samples to characterise the true classification performance on new data.

A similar picture is seen in relation to gene set stability as outlined in Table 3.

**TABLE 3:** Gene filter impact on feature stability.

	eNET		L1-SVM		RF	
Filter	Avg. No. Selected	Stability	Avg. No. Selected	Stability	Avg. No. Selected	Stability
***IBD***						
None	27.8	0.470	35.6	0.491	30	0.835
Low Count	27.9	**0.474**	24.7	0.545	30	**0.841**
Outlier	27.9	0.449	28.4	**0.559**	30	0.833
Low Count + Outlier	27.7	0.458	25.0	0.548	30	0.839
***Sepsis 1***						
None	11.6	0.581	6.400	0.479	10	0.410
Low Count	9.7	0.548	5.646	**0.549**	10	0.462
Outlier	9.7	**0.583**	6.121	0.510	10	0.430
Low Count + Outlier	9.7	0.547	5.485	0.544	10	**0.485**
***Sepsis 2***						
None	27.6	0.369	26.8	0.278	30	0.552
Low Count	25.3	**0.426**	22.7	**0.392**	30	0.588
Outlier	27.2	0.377	27.8	0.268	30	0.562
Low Count + Outlier	25.0	0.424	21.9	0.382	30	**0.603**

The bold values are the highest scoring filter for each dataset.

For the L1-SVM and RF classifiers, feature set stability is shown increase in all three datasets when genes are filtered prior to model training. This is particularly clear for the low counts filter. The stability of the genes selected by eNet show conflicting results, with stability decreasing with the application of the outlier filter in the IBD dataset, and with the application of the low count filter in the Sepsis 1 dataset. The overall trend however is that filtering out uninformative genes results in gene sets that are less sensitive to changes in the data, and therefore likely to be more generalisable to new patient populations.

Beyond the improvements to classification performance and feature stability, filtering to remove uninformative and potentially biasing genes has reduced the dimensionality of the dataset. This has the further benefits of reducing data storage requirements and leading to a simpler optimisation of the model parameters, reducing the computational time required for analysis.

### 3.4 Correspondence with known sepsis biomarkers

A final evaluation of the impact of gene filtering is to determine the consistency of the genes identified by ML gene selection with known biomarkers for sepsis. Here, we compare the top 20 most frequently selected genes over the 100 random sub-samples of the Sepsis 2 dataset to each of three validated transcriptomic biomarker signatures. The signatures used are the Sep3 signature identified by Smith et al. (2014), the 12 gene signature used to compute the “Sepsis Meta Score” (SMS) reported by Sweeney et al. (2015) and the 19 gene “Extended Signature” used to derive the quantitative sepsis response signature (SRSq) reported by Cano-Gamez et al. (2022). A preliminary check is performed to determine whether any of the genes in these signatures are filtered out by applying the low count and outlier filters to the datasets. All genes are retained in the filtered dataset, with the exception of MMP9, removed on the basis of an outlier read count.

Given the small sample sizes and imbalanced nature of the neonatal sepsis datasets, any significance or inference from these investigations with regard new biomarkers cannot be made. However, the correlation between the biomarkers identified before and after gene filtering with validated sepsis biomarkers provides a proxy for biological relevance of the selected genes, and how this is impacted by gene filtering. The Pearson correlation coefficient between all pairs of genes in the normalised and unfiltered data set is calculated. The mean correlation between the top 20 genes identified by each classifier using unfiltered and filtered data, and the genes in the three signatures is calculated for each classifier gene signature pair. The results are shown in Table 4, and heat maps illustrating the individual correlations are provided Supplementary Figures S2—S4.

**TABLE 4:** Mean correlation between genes identified and known sepsis biomarker signatures.

Gene signature	Sep3			Sepsis meta score			SRSq-extended		
Filter	eNet	L1-SVM	RF	eNet	L1-SVM	RF	eNet	L1-SVM	RF
None	0.319	0.279	0.432	0.404	0.351	0.52	0.270	0.232	0.336
Low Count + Outlier	0.340	0.322	0.415	0.408	0.397	0.51	0.276	0.294	0.326
Difference	0.021	0.043	(0.017)	0.004	0.046	(0.010)	0.006	0.062	(0.010)

Both the eNet and L1-SVM classifiers select genes that are more highly correlated with the three known sepsis biomarker signatures after gene filtering; the difference is marginal for eNet but notably different for L1-SVM. These results are consistent with the above observation that the eNet and L1-SVM classifiers selected a significant proportion of uninformative low count genes when applied to the unfiltered dataset. Gene filtering improved classification performance, the stability of the selected genes, and the correlation of the selected genes with known biomarkers. In the case of RF, the average correlations of the identified genes with known biomarkers are lower after gene filtering, consistent with the observations above RF is more robust to low count genes in particular, and material improvements in performance are not observed when gene filtering is applied.

## 4 Conclusion

Biological data is noisy. RNA-Seq count data contains multiple sources of technical and biological noise that can bias ML classification algorithms used in biomarker discovery, resulting in misleading conclusions. In this short study, we have empirically demonstrated the value of independent filtering of genes in RNA-Seq data sets prior to ML gene selection. We have shown that three popular ML algorithms used for gene selection, eNet, L1-SVM, and RF all selected low count and influential outlier genes without filtering first being applied in a range of experimental datasets. We have also demonstrated that gene filtering can improve the classification performance and stability of the resulting gene signature, despite eliminating over 50% of the initial variables. In the case of L1-SVM, we have demonstrated that classification performance is especially improved by employing a low counts filter. Filtering out unnecessary genes brings the additional benefit of working with smaller data sets, reducing computational time and data storage requirements. We also find in the data sets analysed in this study that RF is more robust with small sample sizes to the presence of low counts genes, although this requires further validation to assess generality. In the light of these findings, we offer a number of guidelines on gene filtering to researchers using ML for sepsis biomarker discovery using RNA-Seq data.

1. The performance of gene selection models is very likely to be improved by removing technical and biological stochastic noise from RNA-Seq data, such as very low and outlier read counts. A systematic objective approach should be adopted to filter transcripts to remove such effects, identifying as best as possible the boundary between noise and true biological signal in preference to applying arbitrary thresholds.
2. Include feature set stability in the evaluation metrics for any biomarker discovery analysis. Use stability improvements as well as classification performance to refine gene filtering thresholds and approaches.
3. Compare genes that are filtered out with validated biomarkers for the condition of interest and use this to sense check filter thresholds.
4. In noisy datasets, and where gene filtering appears to remove potentially relevant biomarkers, employ classifiers such as Random Forest, more likely to be robust to the noise in the data

A number of areas are identified for further work. Firstly, this work only considers a single approach for low count gene filtering and influential outlier detection. Multiple other methodologies exist in the literature, and further work is needed to evaluate their impact on ML classification performance, and how best to set a filter threshold in the context of ML gene selection analysis. Secondly, the impact of additional filters, whether to reduce other sources of technical noise and bias beyond low and outlier counts, and gene filters based on biological criteria should be investigated for their impact on ML performance. Thirdly, the work should be extended to a broader range of ML classifiers and datasets to be able to draw firmer conclusions on the level of gene filtering required for a specific class of classifier. Finally, in the case of sepsis biomarker discovery, a broader patient population is required for analysis to be able to confidently declare novel discriminate biomarkers for sepsis using the approaches described here.

## Data Availability

The IBD dataset can be found in the EMBL-EBI ArrayExpress database (E-MTAB-11349). The Sepsis 1 dataset can be found in the NCBI GEO Omnibus database (GSE138712). A copy of the Sepsis 2 dataset, with transcripts and patients de-identified can be found at https://github.com/parkyed/SepsisClassifiers. The analysis is implemented in R and the code is freely available at https://github.com/parkyed/SepsisClassifiers.

## Footnotes

1 Given the same cross validation scheme is used to both select the hyperparameter values that provide the desired number of genes and to evaluate classification performance, it is acknowledged the performance estimate is biased as a result, however given the low sample sizes of the Neonatal Sepsis datasets, a hold out test set is deemed impractical

## Abbreviations

ML, Machine Learning; IBD, Inflammatory Bowel Disease; PAX, Potassium Amyl Xanthate; GLM, Generalised Linear Model; eNet, Elastic Net Regularised Logistic Regression; L1-SVM, L1 penalised Support Vector Machine; RF, Random Forest; PPV, Positive Predictive Value.
