# Supplementary material for: Gene filtering strategies for machine learning guided biomarker discovery using neonatal sepsis RNA-seq data
Source: Front Genet. 2023 Apr 11;14:1158352. doi: 10.3389/fgene.2023.1158352 (PMC10126415; doi:10.3389/fgene.2023.1158352)
Supplement: Supplementary file 1 [file Image1.pdf]

Supplementary Material

1 SUPPLEMENTARY FIGURES

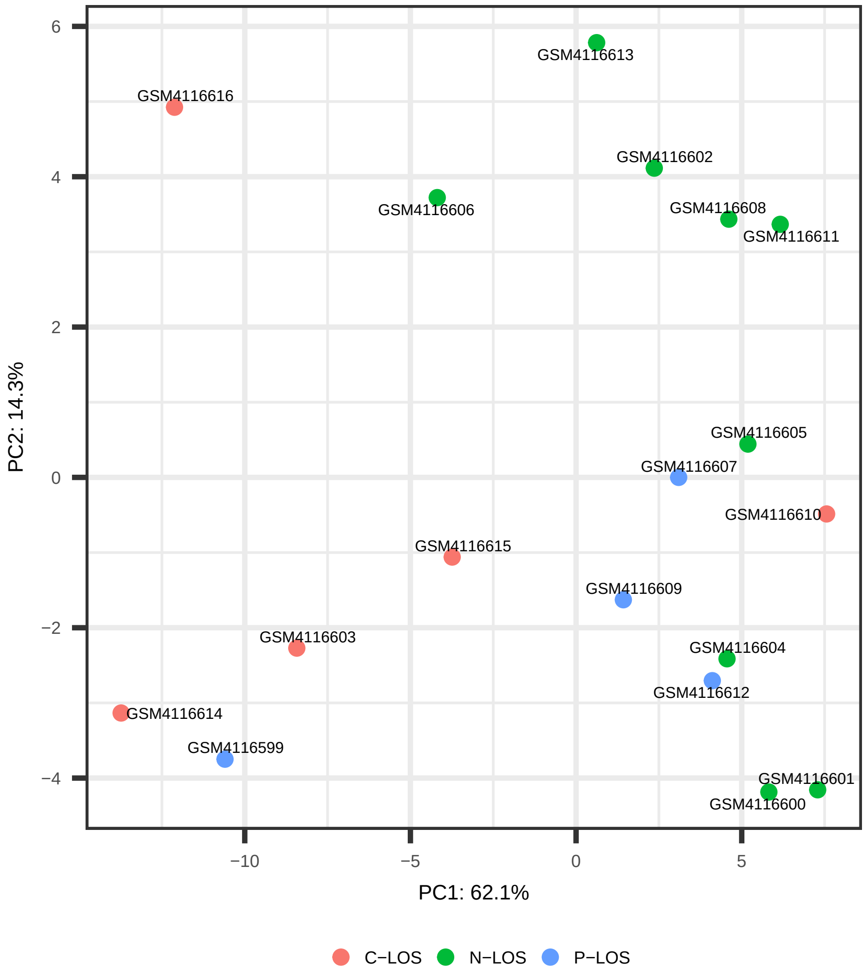

Figure S1. PCA analysis of Protect dataset using genes in the 52-gene signature

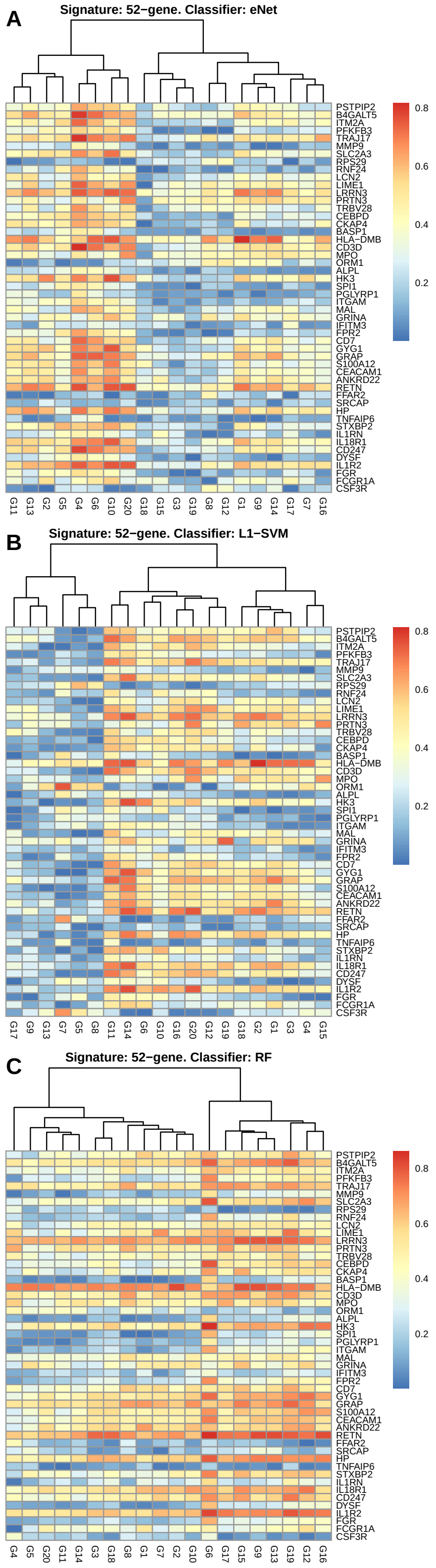

Figure S2. Pearson correlation coefficient between normalised read counts for genes in the 52-gene signature and the top 20 genes selected in the Protect dataset using **(A)** eNet, **(B)** L1-SVM and **(C)** RF classifiers

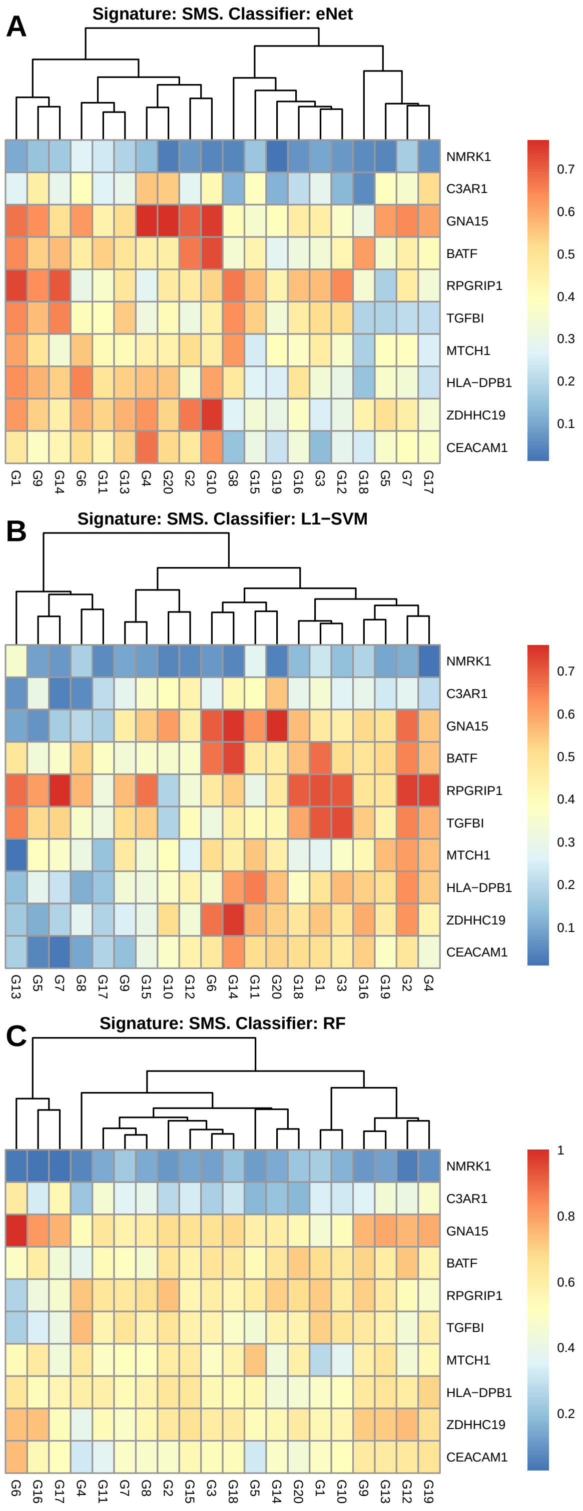

Figure S3. Pearson correlation coefficient between normalised read counts for the SMS signature and the top 20 genes selected in the Protect dataset using **(A)** eNet, **(B)** L1-SVM and **(C)** RF classifiers

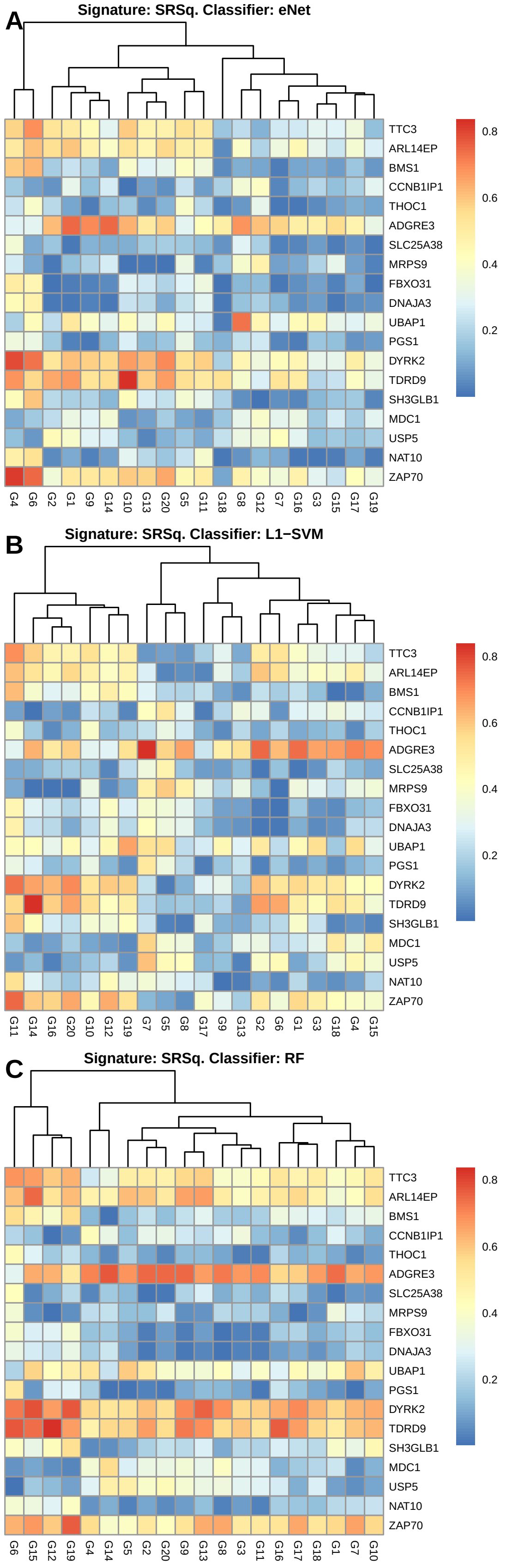

Figure S4. Pearson correlation coefficient between normalised read counts for genes in the SRSq signature and the top 20 genes selected in the Protect dataset using **(A)** eNet, **(B)** L1-SVM and **(C)** RF classifiers
